# Enterobiasis and strongyloidiasis and associated co-infections and morbidity markers in infants, preschool- and school-aged children from rural coastal Tanzania: a cross-sectional study

**DOI:** 10.1186/s12879-014-0644-7

**Published:** 2014-12-09

**Authors:** Nahya Salim, Tobias Schindler, Ummi Abdul, Julian Rothen, Blaise Genton, Omar Lweno, Alisa S Mohammed, John Masimba, Denis Kwaba, Salim Abdulla, Marcel Tanner, Claudia Daubenberger, Stefanie Knopp

**Affiliations:** Bagamoyo Research and Training Centre, Ifakara Health Institute, Bagamoyo, Tanzania; Department of Pediatrics and Child Health, Muhimbili University Health and Allied Sciences, Dar es Salaam, Tanzania; Department of Epidemiology and Public Health, Swiss Tropical and Public Health Institute, Basel, Switzerland; University of Basel, Basel, Switzerland; Department of Medical Parasitology and Infection Biology, Swiss Tropical and Public Health Institute, Basel, Switzerland; Department of Ambulatory Care and Community Medicine, Infectious Disease Service, Lausanne University Hospital, Lausanne, Switzerland; Department of Life Sciences, Wolfson Wellcome Biomedical Laboratories, Natural History Museum, London, United Kingdom

**Keywords:** Asymptomatic Plasmodium parasitaemia, Anaemia, Anthropometric measures, Co-infection, Enterobius vermicularis, Haematology, Hookworm, Soil-transmitted helminths, Strongyloides stercoralis, Tanzania, Trichuris trichiura

## Abstract

**Background:**

There is a paucity of data pertaining to the epidemiology and public health impact of *Enterobius vermicularis* and *Strongyloides stercoralis* infections. We aimed to determine the extent of enterobiasis, strongyloidiasis, and other helminth infections and their association with asymptomatic *Plasmodium* parasitaemia, anaemia, nutritional status, and blood cell counts in infants, preschool-aged (PSAC), and school-aged children (SAC) from rural coastal Tanzania.

**Methods:**

A total of 1,033 children were included in a cross-sectional study implemented in the Bagamoyo district in 2011/2012. Faecal samples were examined for intestinal helminth infections using a broad set of quality controlled methods. Finger-prick blood samples were subjected to filariasis and *Plasmodium* parasitaemia testing and full blood cell count examination. Weight, length/height, and/or mid-upper arm circumference were measured and the nutritional status determined in accordance with age.

**Results:**

*E. vermicularis* infections were found in 4.2% of infants, 16.7%, of PSAC, and 26.3% of SAC. *S. stercoralis* infections were detected in 5.8%, 7.5%, and 7.1% of infants, PSAC, and SAC, respectively. Multivariable regression analyses revealed higher odds of enterobiasis in children of all age-groups with a reported anthelminthic treatment history over the past six months (odds ratio (OR): 2.15; 95% confidence interval (CI): 1.22 - 3.79) and in SAC with a higher temperature (OR: 2.21; CI: 1.13 - 4.33). Strongyloidiasis was associated with eosinophilia (OR: 2.04; CI: 1.20-3.48) and with *Trichuris trichiura* infections (OR: 4.13; CI: 1.04-16.52) in children of all age-groups, and with asymptomatic *Plasmodium* parasitaemia (OR: 13.03; CI: 1.34 - 127.23) in infants. None of the investigated helminthiases impacted significantly on the nutritional status and anaemia, but moderate asymptomatic *Plasmodium* parasitaemia was a strong predictor for anaemia in children aged older than two years (OR: 2.69; 95% CI: 1.23 – 5.86).

**Conclusions:**

*E. vermicularis* and *S. stercoralis* infections were moderately prevalent in children from rural coastal Tanzania. Our data can contribute to inform yet missing global burden of disease and prevalence estimates for strongyloidiasis and enterobiasis. The association between *S stercoralis* and asymptomatic *Plasmodium* parasitaemia found here warrants further comprehensive investigations.

**Electronic supplementary material:**

The online version of this article (doi:10.1186/s12879-014-0644-7) contains supplementary material, which is available to authorized users.

## Background

Soil-transmitted helminth infections belong to the neglected tropical diseases [1]. The most common soil-transmitted helminths are roundworms (*Ascaris lumbricoides*), whipworms (*Trichuris trichiura*), and hookworms (*Ancylostoma duodenale* and *Necator americanus*). Infections are acquired via the ingestion of the parasites’ eggs, for example with contaminated food, or in case of hookworms via larvae penetrating bare skin [2],[3]. The adult worms live in the intestines of humans, and eggs or larvae are excreted with faeces and thrive in the soil. Hence, infections are particularly prevalent in areas, where hygiene and sanitation are low and where the environment favours a rapid transmission. It is estimated that globally around 1.45 billion people are infected with at least one intestinal helminth species, and a global burden of 5.2 million disability adjusted life years (DALYs) is attributed to these infections [4]-[6].

In addition to the four major soil-transmitted helminth species, there are two intestinal nematodes infecting humans, the threadworm *Strongyloides stercoralis* and the pinworm *Enterobius vermicularis* that are mostly neglected in prevalence reports and global burden of disease estimates due to their rather unpleasant and cumbersome diagnosis with the Baermann and/or Koga agar plate methods or adhesive tape test, respectively, and due to difficulties to assess associated morbidity [1],[2],[7]-[10].

*S. stercoralis* has an intra- and extra-human lifecycle during which the parasite can reproduce asexually and sexually, respectively. Larvae that hatched from eggs within the intestines are able to penetrate the intestinal wall and can perpetuate the lifecycle within the human host for decades after initial exposure [2],[11]. Infections are mostly mild and often asymptomatic in otherwise healthy individuals [12]. Skin lesions, pulmonary and gastro-intestinal symptoms, and blood eosinophilia are reported as unspecific disease markers [10],[13]-[17]. Chronically infected immunocompromised patients, however, are at high risk of developing a lethal hyper-infection syndrome, caused by proliferating tissue-invasive larvae that might carry bacteria from the intestines to organs, leading to systemic infections, multiorgan failure and systemic sepsis [1],[12],[18]. It is estimated that *S. stercoralis* affects between 10% and 40% of the population in tropical- and sub-tropical countries, but adequate information is lacking, particularly for the high-risk areas including Sub-Saharan Africa and Southeast Asia [19]. Global burden of disease estimates do not exist for this parasite [5],[19],[20] and strongyloidiasis remains an underestimated health problem [21]. Good reliable data on infection, morbidity, and indisputable causal links between them, are urgently needed to make the public health argument for a guided response against *S. stercoralis* [7].

*E. vermicularis* is transmitted via the ingestion of eggs contained in dust, water, or sticking on hands and food. The female adults live in the cecum and large intestines and migrate to the anus to deposit their eggs on the perianal skin [2]. Since eggs become infective within hours, autoinfection is possible [22]. Enterobiasis includes symptoms such as intense pruritus in the perianal area, which can lead to insomnia, restlessness, and irritability. Moreover, the adult pinworms can migrate into the appendix or genital tract, causing appendicitis and genitourinary complications [23],[24]. Ectopic infections of liver, lung, kidneys, and other organs occur infrequently [24],[25]. Enterobiasis is considered the most common helminth infection worldwide [26]. It is estimated that globally 4-28% of children are infected [3], but recent and profound prevalence and burden estimates are missing, particularly for Sub-Saharan Africa.

The neglect of strongyloidiasis and enterobiasis in most studies pertaining to soil-transmitted helminth infections results in a knowledge gap about their potential impact on co-infections and the nutritional status of the host. While it is widely acknowledged that helminth infections modulate immune responses and might influence the acquisition and outcomes of diseases such as malaria, tuberculosis (TB), and human immunodeficiency virus-acquired immune deficiency syndrome (HIV-AIDS), the exact mechanisms are not known [27]-[37]. Research on this topic has gained much interest over the past years, but results are inconsistent. Multiple dimensions seem to be involved, including helminth species, infection intensity, and the host’s age, genetics, and immunological and nutritional status [38],[39].

The IDEA research program is designed to intensively study the immunological interplay between helminth infections and malaria, TB, and HIV-AIDS (http://ec.europa.eu/research/health/infectious-diseases/neglected-diseases/projects/014_en.html). Of course, to obtain reliable results, not only the presence of major helminth species, but also infections with *S. stercoralis* and *E. vermicularis* need to be taken into account. Here, we present, to our knowledge for the first time, the extend of strongyloidiasis and enterobiasis, besides other helminthiases, in infants, preschool-aged children (PSAC), and school-aged children (SAC) from rural coastal Tanzania that participated in a cross-sectional study implemented to recruit participants for the IDEA-malaria study arm in the Bagamoyo district, United Republic of Tanzania. We also show anthropometric and haematological characteristics of the study group and associations found between the individual helminth species infections, between helminth infections and asymptomatic *Plasmodium* infections and between helminth infections and anthropometric status, anaemia, and additional haematological parameters in different age-groups. With this report we aim to shed more light on the distribution and public health importance of two highly neglected soil-transmitted helminth species and therefore to contribute to inform yet missing global burden and prevalence estimates.

## Methods

### Ethics statement

The protocol for the IDEA project conducted at the Bagamoyo Research and Training Center of the Ifakara Health Institute (IHI-BRTC) in Bagamoyo, United Republic of Tanzania, was approved by the institutional research commissions of the Swiss Tropical and Public Health Institute (Swiss TPH; Basel, Switzerland) and the IHI. Ethical approval for the study was obtained from the Ethikkomission beider Basel (EKBB; Basel, Switzerland; reference number: 257/08) and the National Institution for Medical Research of Tanzania (NIMR; Dar es Salaam, United Republic of Tanzania; reference number: NIMR/HQ/R.8a/Vol.IX/1098). The study was monitored by the IHI internal monitoring team in collaboration with the Pan African Collaboration for the Evaluation of Antituberculosis Antibiotics (PanACEA) and Swiss TPH.

Local district, community, school, and health authorities were informed about the purpose and procedures of the study and their acceptance for the implementation was sought. Individuals eligible to be screened and to potentially participate in one of the study arms of IDEA were informed in detail about the aims of the study, the risks and benefits of study participation, and the extend of time involvement in case of their participation [9]. It was pointed out that participation was voluntary and that participants could withdraw from the study at any time without further obligation. All adult participants and in case of children aged ten years or younger (ten years being the age-limit of children eligible for inclusion in the IDEA-malaria study arm) their parents or legal guardians, were asked to sign a written informed consent sheet if they agreed to participate. Participants’ privacy was preserved by de-identification of the dataset. Participants’ names were replaced by individual identification codes and all information pertaining to names, geographical locations, and dates of birth, sample collection, and sample examination was removed from the dataset.

Patients with helminthiases, malaria, TB, HIV-AIDS, and/or other medical conditions received treatment according to the national treatment guidelines of the United Republic of Tanzania. Accordingly, participants of our cross-sectional survey that were infected with helminths were treated with albendazole (400 mg single oral dose) against common soil-transmitted helminth infections, *S. stercoralis* and *E. vermicularis*, or praziquantel (40 mg/kg) against *Schistosoma* infections. Participants with asymptomatic *Plasmodium* parasitaemia or clinical malaria were treated with Artemether Lumefantrine and children with severe malaria received Quinine.

### Study area

All participants included in the present analysis resided in one among 12 villages or small towns in the Bagamoyo district, belonging to the Pwani region in the United Republic of Tanzania. These settlements are located in a rural environment approximately 20-60 kilometres from Bagamoyo, which is a historical town located directly at the coast along the Indian Ocean, 75 kilometres north of Dar es Salaam. The population of the Bagamoyo district was estimated at 311,740 inhabitants in the 2012 census [40]. The IHI-BRTC, where the laboratory work was conducted, is located right beside the Bagamoyo District Hospital in the heart of the town.

The average temperature for the region is 28°C and there are two annual rainy seasons, the heavy Masika rains from March to June and the light Vuli rains from October to December. The leading source of income of the region is cash crop production of coconuts, fruits and cashew nuts, livestock farming and, yet to a lesser extent, fishing and salt mining [41]. The inhabitants of the region are mainly smallholder farmers engaged in food crop production such as vegetables, cassava, pulses, maize, and paddy [41].

### Study design and participant recruitment

The IDEA study is designed as longitudinal short-term study with three study arms [9]. In the present study, we focused on results of a cross-sectional study, implemented to recruit children aged two months to ten years with asymptomatic *Plasmodium* parasitaemia for the IDEA-malaria study arm. In that arm, a sample size of 100 children with asymptomatic *Plasmodium* parasitaemia was required. Expecting a prevalence of 10% of asymptomatic *Plasmodium* parasitaemia (as found in previous, yet unpublished studies in the Bagamoyo area), we had to enrol about ~1,000 children living in the Bagamoyo district in the cross-sectional study. The fieldwork for recruitment and data collection started in early 2011 and was concluded in November 2012.

### Field procedures

Before the onset of the study, sensitization and information meetings were hold with community leaders, village health care workers (VHCW), teachers, and local clinicians. Parents were informed by VHCW about the study and asked to bring the children that they considered as healthy to meeting points to participate in the health screening to recruit children into the asymptomatic IDEA-malaria study arm on a specific day. At the meeting point, the study aims and procedures were explained in detail and in lay terms to the children and their accompanying parent or legal guardian. If the patient or parent/legal guardian orally assented to participation, the parent/legal guardian was given an information sheet and asked to provide a written informed consent for the child to participate. In case of illiteracy, a thumbprint signature was obtained.

Upon submission of the signed consent sheet, the participant’s demographics, vitals, clinical signs and symptoms, and reported anthelmintic treatment history (treatment with albendazole or mebendazole within the past 6 months) and use of bed nets were entered in standardized forms. Children’s axillary temperature, height or length (measured with standardized height/length boards from the World Health Organization (WHO)/United Nations Children’s Fund (UNICEF)), weight (measured with a 25 kilogram hanging scale (C. M. S. Weighing Equipment Ltd., London, United Kingdom) for light children and with a seca weighing scale (seca Deutschland, Hamburg, Germany) for heavier children), and mid-upper arm circumference (MUAC; measured with the standard UNICEF MUAC tape (S0145620 MUAC, Child 11.5 Red/PAC-50)) were recorded. A finger-prick blood sample (1.0 ml) was collected and stored on ice immediately after collection until examination in the laboratory at IHI-BRTC. Each participant was provided with (i) two plastic containers (100 ml) with lid for urine and stool collection, respectively, and (ii) an adhesive tape (50 × 20 mm) and a microscope slide, all labelled with an individual identifier code. The participants were invited to collect an own urine and morning stool sample of sufficient size (i.e. to fill half of the container) to perform all laboratory examinations. Additionally, they were instructed to wash their buttocks before going to bed and, in the morning after wake up and before taking a shower, to apply the adhesive tape on their anus before sticking it to the microscope slide. The filled containers and adhesive tape slides were collected by a VHCW the next day around noon and transferred to the Helminth Unit laboratory of the IHI-BRTC, where all stool and urine samples were examined.

### Laboratory procedures

The blood samples were examined for a full blood cell count including a differential white blood cell count using an externally quality controlled Sysmex XS-800 Haematology Analyser (Sysmex Deutschland GmbH, Norderstedt, Germany). Malaria parasitaemia was assessed using the SD Bioline Malaria Ag Pf/Pan 05FK60 rapid test (Standard Diagnostics, Kyonggi, Republic of Korea) and by microscopy. For the latter purpose, two thick blood smear slides (using 6 μl of blood) per patient were prepared, stained with Giemsa, and examined by two certified malaria microscopists who independently counted the number of asexual *Plasmodium* parasite stages per up to 500 white blood cells counts, depending on the level of parasitaemia [42]. *Plasmodium* species were not differentiated, but it is widely assumed that *P. falciparum* is the major cause of malaria in Tanzania [43]. Blood samples from children who entered the study from July 2012 onwards were additionally examined for *Wuchereria bancrofti* antigen using the Binax NOW Filariasis rapid immunochromatic test (ICT)-card (Inverness Medical Professional Diagnostics; Scarborough, ME, United States of America). In brief and according to the manufacturer’s manual, 100 μl of whole blood were transferred on the ICT-card. Subsequently, the test card was closed and incubated for 10 min before the result was read.

Stool and urine samples were processed by experienced laboratory technicians as described in detail elsewhere [9]. Firstly, the Baermann method was applied for the detection of *S. stercoralis* larvae [44]. Secondly, during the Baermann incubation time, from the remainder of the stool sample of each participant duplicate Kato-Katz thick smear slides were prepared using a 41.7 mg template [45] for the diagnosis and number of *A. lumbricoides*, hookworm, *T. trichiura* and *S. mansoni* eggs. Thirdly, adhesive tape slides were microscopically examined for the presence of *E. vermicularis* eggs. Fourthly, the urine sample of each participant was investigated for microhaematuria using a dipstick (Haemastix; Siemens Healthcare Diagnostics, Eschborn, Germany) and for *S. haematobium* eggs by duplicate urine filtration slides (hydrophilic polycarbonate membrane filter; pore size 20 micron, diameter 13 mm; Sterlitech, Kent, WA, United States of America) [46]. Fifthly, the following morning, before new samples arrived, a subsample of each individual’s stool (~1 g), which had been preserved in sodium acetate acetic acid formalin (SAF) over night, was examined for the presence and number of helminth eggs with the FLOTAC dual technique [47] using flotation solution 2 (FS2; saturated sodium chloride (NaCl) solution; specific gravity (s.g.): 1.20) and FS7 (zinc sulfate (ZnSO4 7H2O) solution; s.g.: 1.35). At least 10% of the blood smear, Kato-Katz thick smear, adhesive tape and urine filtration slides were subjected to re-examination for quality control by independent trained microscopists blinded to the initial result.

### Data management and statistical analysis

Each participant’s data were entered manually in the participant’s case report form (CRF), subsequently transferred into an electronic data base (Microsoft Access 2010, Microsoft Corporation; Redmont, WA, United States of America; and DMSys, SigmaSoft International; Chicago, IL, United States of America) and finally transferred, cleaned, and analysed using STATA version 9.2 (StataCorp.; College Station, TX, United States of America). Anthropometric z-scores were calculated using the WHO Anthro and AnthroPlus softwares (WHO; Geneva, Switzerland).

Children were grouped according to their age into infants (0-2 years), PSAC (3-4 years), and SAC (5-10 years). According to WHO guidelines, moderate and severe malnutrition were defined at a MUAC cut-off of 12.5 cm and 11.5 cm, respectively, for infants and PSAC [48],[49]. Moderate and severe wasting were defined by applying a weight-for-height z-score cut-off below -2 and -3, respectively, for infants and PSAC and for combined sexes [49]. The weight-for-height of infants whose length was under 45 cm was not calculated. In the same age-group, moderate and severe underweight were defined at height-for-age z-scores of -2 and -3, respectively. For SAC, thinness and severe thinness were defined by a body mass index (BMI)-for-age z-score cut-off below -2 and -3, respectively, and stunting at a height-for-age z-score cut-off below -2.

Anaemia thresholds were applied according to WHO recommendations as follows: haemoglobin values >10.9 mg/l non-anaemia, 10.0-10.9 mg/l mild anaemia, 7.0-9.9 mg/l moderate anaemia, and <7.0 mg/l severe anaemia for infants and PSAC, and haemoglobin values >11.5 mg/l non-anaemia, 11.0-11.4 mg/l mild anaemia, 8.0-10.9 mg/l moderate anaemia, and <8.0 mg/l severe anaemia for SAC [50]. Reference values for the full blood cell counts were taken from Buchanan and colleagues (2004) who provide haematology reference values for healthy Tanzanian children from the Kilimanjaro region stratified by age, including infants, PSAC, and SAC [51]. The cell counts of our children were classified as low, when they were below the 95% confidence interval (CI) limit and as high when they were above the 95% CI limit of the Kilimanjaro reference group. Fever was considered as axillary temperature of >38.0°C as suggested by the Brighton Collaboration [52].

A patient was considered to be infected with a helminth species, if the infection was detected with one or several diagnostic methods. For each individual, helminth infection intensity was determined according to Kato-Katz thick smear results as suggested by the WHO [53]. For this purpose, faecal egg counts (FEC) as recorded from each Kato-Katz thick smear microscopic examination were transferred into eggs per gram of stool (EPG) by multiplying the average FEC from duplicate Kato-Katz thick smears of each individual by a factor 24. The lower limits of moderate and heavy infections were 5,000 and 50,000 EPG for *A. lumbricoides*, 1,000 and 10,000 EPG for *T. trichiura*, 2,000 and 4,000 EPG for hookworm and 99 and 399 EPG for *S. mansoni*, respectively. Microhaematuria was classified according to the manufacturer’s suggestion into negative, trace, +, ++, or +++ and *S. haematobium* egg counts into light (1–49 eggs/10 ml of urine) and heavy (≥50 eggs/10 ml of urine). Asymptomatic *Plasmodium* parasitaemia was defined by a positive malaria rapid diagnostic test result and/or by *Plasmodium* parasites detected microscopically plus the absence of unspecific symptoms of malaria at the day of enrolment or over the past seven days (i.e., fever, flue, cough, difficult breathing, and/or abdominal discomfort). Counts of below 10 parasites per 200 white blood cells (i.e. less than 400 parasites per μl blood) were defined as low grade parasitaemia, and counts of 100 and more parasites per 200 white blood cells (i.e. 400 or more parasites per μl blood) as moderate parasitaemia.

To assess a direct interaction between helminth and helminth co-infections, and between helminth and asymptomatic *Plasmodium* parasitaemia co-infections we calculated observed and expected prevalences for co-infections [54]. The expected co-infection prevalences were calculated as the product of the observed prevalence of one infection (regardless of a co-infection) and the observed prevalence of the second infection (regardless of a co-infection). For comparison of the observed *versus* expected prevalences, the Fisher’s exact test (two-sided) was applied.

Multivariable logistic regression analyses were used for estimating odds ratios (ORs), including 95% CIs, to determine associations between different helminth species infections or asymptomatic *Plasmodium* parasitaemia or anaemia (binary outcome variables), and nutritional measures (ordinal or binary explanatory variable), anaemia (ordinal or binary explanatory variable), specific full blood cell count variables including haemoglobin (continuous explanatory variable), helminth co-infections (binary explanatory variable), asymptomatic *Plasmodium* parasitaemia (binary explanatory variable), temperature (continuous explanatory variable), or fever (binary explanatory variable). In all multivariable analyses we adjusted for age in months (continuous explanatory variable), sex (binary explanatory variable), and reported anthelmintic treatment in the past six months (binary explanatory variable) and included any significant explanatory variable from univariable models pertaining to the same outcome, excluding co-linear variables. For the multivariable logistic regression, we applied a backward stepwise procedure removing non-predicting covariates up to a significance level of 0.2 and allowed for possible clustering within houses by using the sandwich estimator robust cluster option in STATA. Both, univariable and multivariable regression analyses were run (i) for all ages and (ii) stratified by age-group.

## Results

### Study group

**Figure 1:**
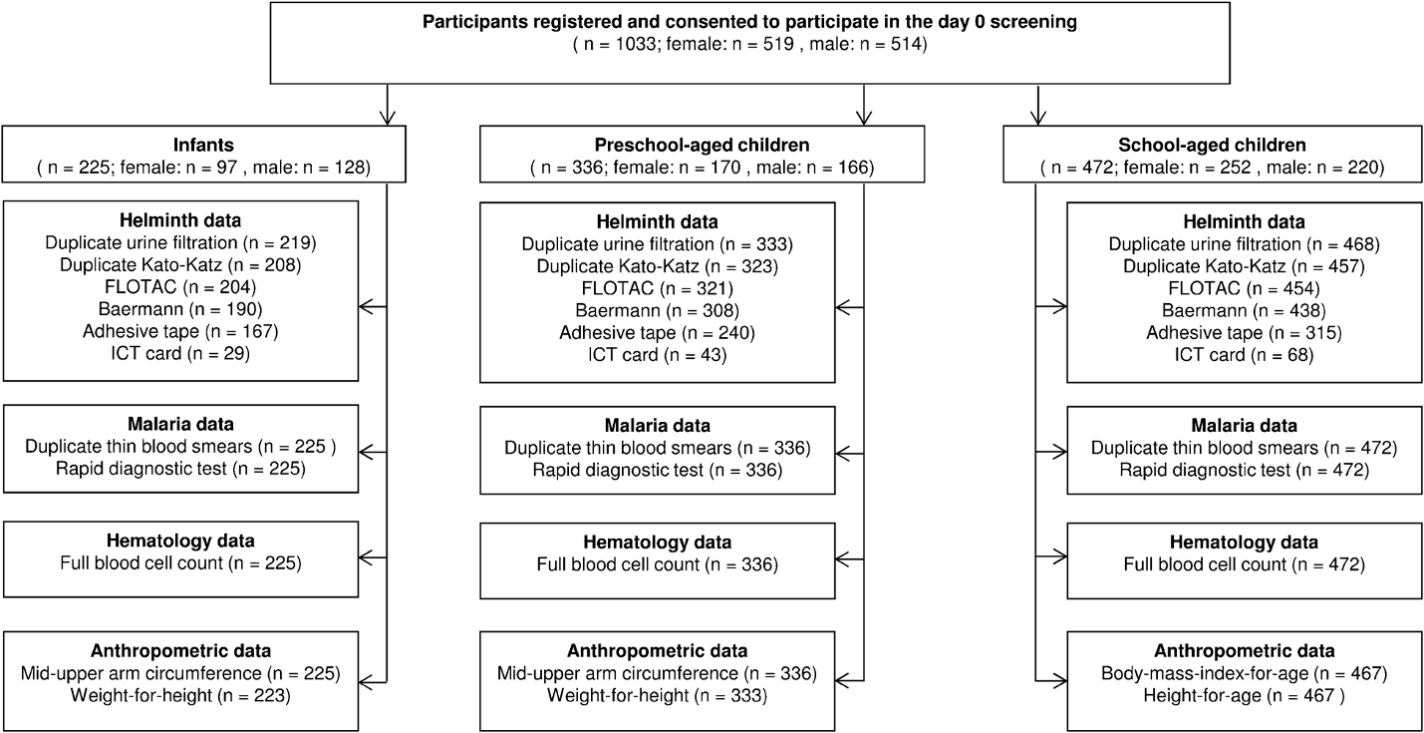
**Characteristics of the IDEA-** **malaria study group consisting of children from the Bagamoyo district,** **United Republic of Tanzania.**

Written informed consent to participate in the cross-sectional survey was provided for 1,033 children. Among them, 519 were girls and 514 were boys. According to their month and year of birth, 225 were grouped as infants, 336 as PSAC and 472 as SAC. The numbers of children with parasitological, haematological, and anthropometric examinations in each age-group are shown in Figure 1.

### Anthropometric and haematological characteristics

In our study group, 1.3% of infants were wasted and 2.2% were underweight (Table 1). Among the PSAC, 0.3% were wasted and 2.4% were underweight. Thinness and stunting were detected in 3.2% and 18.7% of SAC, respectively. Anaemia was observed in 85.6%, 47.3%, and 45.6% of infants, PSAC, and SAC, respectively.

**Table 1:** **Anthropometric and anaemia status of infants**, **preschool**-**aged** (**PSAC**), **and school**-**aged children** (**SAC**) **from the Bagamoyo district**, **United Republic of Tanzania**, **calculated in line with guidelines and thresholds provided by the World Health Organization** [48]-[50]

	Age group								
	Infants			PSAC			SAC		
Anthropometric aspects	Total	n	%	Total	n	%	Total	n	%
**Mid-** **upper arm circumference**	225			336					
Normal		222	98.7		335	99.7			
Moderately wasted		3	1.3		1	0.3			
Severely wasted		0	0		0	0			
**Weight-** **for-** **height**	223			333					
Normal		218	97.8		325	97.6			
Moderate underweight		2	0.9		7	2.1			
Severe underweight		3	1.3		1	0.3			
**Body-** **mass-** **index-** **for** **-age**							467		
Normal								452	96.8
Moderate thinness								10	2.1
Severe thinness								5	1.1
**Height-** **for-** **age**							467		
Normal								380	81.4
Moderate stunting								68	14.6
Severe stunting								19	4.1
**Anaemia**	221			334			471		
Normal		32	14.5		176	52.7		256	54.4
Mild anaemia		74	33.5		108	32.3		81	17.2
Moderate anaemia		106	48.0		47	14.1		127	27.0
Severe anaemia		9	4.1		3	0.9		7	1.5

As shown in Table 2, the full blood cell counts from children belonging to our study group revealed that more than 70% of the children had haematocrit, mean corpuscular volume, platelets, white blood cell counts, neutrophil and lymphocyte counts within the normal range, when compared to haematological reference values derived from children of the same age-groups residing in the Kilimanjaro district, United Republic of Tanzania [51]. However, more than 10% of our children in any age-group had elevated white blood cell and lymphocyte counts and more than 20% had high neutrophil counts. High monocyte, eosinophil and basophil counts were detected in more than 30% of our children regardless of age-group.

**Table 2:** **Haematological values derived from full blood cell counts from infants**, **preschool**-**aged (PSAC)**, **and school**-**aged children (SAC) from the Bagamoyo district**, **United Republic of Tanzania**

Age group	Infants			PSAC			SAC		
	Total	n	%	Total	n	%	Total	n	%
**Haematocrit** **(%)**	225			336			472		
Low		12	5.3		5	1.5		60	12.7
Normal		190	84.4		305	90.8		372	78.8
High		23	10.2		26	7.7		40	8.5
**Mean corpuscular volume** **(pg)**	225			336			472		
Low		19	8.4		2	0.6		28	5.9
Normal		186	82.7		308	91.7		408	86.4
High		20	8.9		26	7.7		36	7.6
**Platelets** **(** **10** ^**9**^ **/l)**	**225**			336			472		
Low		1	0.4		5	1.5		15	3.2
Normal		202	89.8		305	90.8		420	89.0
High		22	9.8		26	7.7		37	7.8
**White blood cells** **(10** ^**9**^ **/l)**	**225**			336			472		
Low		0	0.0		0	0.0		4	0.8
Normal		182	80.9		288	85.7		351	74.4
High		43	19.1		48	14.3		117	24.8
**Neutrophils** **(10** ^**9**^ **/l)**	**225**			336			472		
Low		2	0.9		0	0.0		6	1.3
Normal		176	78.2		266	79.2		350	74.2
High		47	20.9		70	20.8		116	24.6
**Lymphocytes** **(10** ^**9**^ **/l)**	**225**			336			472		
Low		6	2.7		6	1.8		5	1.1
Normal		183	81.3		292	86.9		374	79.2
High		36	16.0		38	11.3		93	19.7
**Monocytes** **(10** ^**9**^ **/l)**	**225**			336			472		
Low		0	0.0		0	0.0		0	0.0
Normal		114	50.7		198	58.9		249	52.8
High		111	49.3		138	41.1		223	47.2
**Eosinophils** **(10** ^**9**^ **/l)**	**225**			336			472		
Low		11	4.9		12	3.6		12	2.5
Normal		127	56.4		187	55.7		276	58.5
High		87	38.7		137	40.8		184	39.0
**Basophils** **(10** ^**9**^ **/l)**	**225**			336			472		
Low		2	0.9		0	0.0		0	0.0
Normal		143	63.6		221	65.8		252	53.4
High		80	35.6		115	34.2		220	46.6

Reference values are derived from children living in the Kilimanjaro district [51].

### Parasitic infections, fever, and microhaematuria

Among all children examined for helminth infections, *E. vermicularis* was found in 18.0%, hookworm in 9.1%, *S. stercoralis* in 6.9%, *T. trichiura* in 2.5%, *W. bancrofti* in 1.4%, *S. haematobium* in 0.3%, and *A. lumbricoides* in 0.1%. No child was diagnosed with a *S. mansoni* infection.

**Figure 2:**
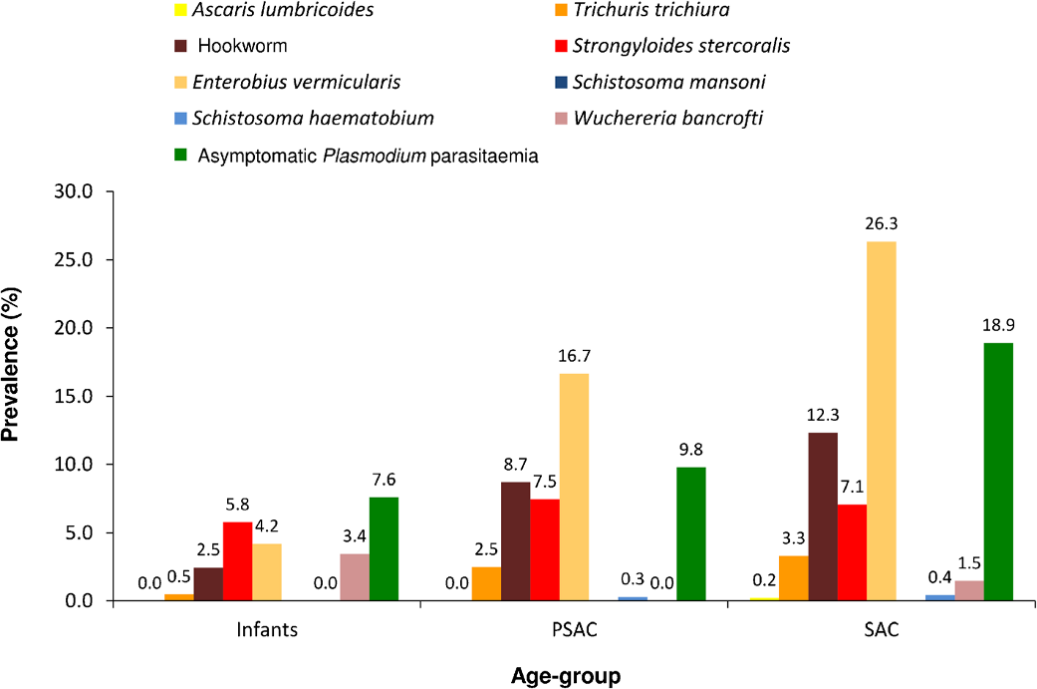
**Prevalence of helminth infections and asymptomatic** ***Plasmodium*** **parasitaemia in infants,** **preschool-** **aged children** **(PSAC)** **and school-** **aged children** **(SAC)** **from the Bagamoyo district,** **United Republic of Tanzania.**

Stratified by age-group, infections with any investigated helminth species were found in 10.2% of infants, 25.0% of PSAC, and 33.5% of SAC. As shown in Figure 2, the most prevalent helminth infections in infants were with *S. stercoralis* (5.8%) and *E. vermicularis* (4.2%), followed by *W. bancrofti* (3.4%), hookworm (2.5%), and *T. trichiura* (0.5%). The youngest children infected with *T. trichiura*, *W. bancrofti*, *S. stercoralis*, *E. vermicularis*, and hookworms were aged six, seven, ten, eleven, and 15 months, respectively. PSAC and SAC were mostly infected with *E. vermicularis* (16.7% and 26.3%, respectively), hookworm (8.7% and 12.3%, respectively), *S. stercoralis* (7.5% and 7.1%, respectively), and *T. trichiura* (2.5% and 3.3%, respectively).

Among the 70 children diagnosed with a hookworm infection by the Kato-Katz method, 60 (85.7%) showed a light, 6 (8.6%) a moderate, and 4 (5.7%) a heavy infection intensity. Among the 21 *T. trichiura* positive children, 19 (90.5%) had a light and 2 (9.5%) a moderate infection intensity. The only child infected with *A. lumbricoides* had a moderate infection intensity. All three children infected with *S. haematobium* had light infection intensities.

Asymptomatic *Plasmodium* parasitaemia was diagnosed in 7.6% of infants, 9.8% of PSAC, and 18.9% of SAC, respectively. Among all children with asymptomatic *Plasmodium* parasitaemia, 66.3% had a moderate parasitaemia and 33.7% had a low parasitaemia. Fever at the day of examination was measured in 1.3% of infants and 0.6% of SAC without *Plasmodium* parasitaemia. Microhaematuria was detected in 10.5% of infants, 3.9% of PSAC, and 2.4% of SAC, respectively.

### Comparison between observed and expected co-infection prevalences

A summary of observed *versus* expected parasite co-infection prevalences is presented in Table 3. Significant differences in prevalence, suggestive for non-chance findings, were detected for co-infection with *S. stercoralis* and asymptomatic *Plasmodium* parasitaemia in children of all age-groups (p = 0.039), but particularly in infants (p = 0.006). Moreover, observed co-infections with *S. stercoralis* and hookworm in all age-groups (p = 0.038), *S. stercoralis* and *T. trichiura* in all age-groups (p = 0.018), but particularly in SAC (p = 0.016), and hookworm and *T. trichiura* in all age-groups (p = 0.004), but particularly in PSAC (p = 0.022) were significantly higher than expected by chance.

**Table 3:** **Infants’**, **preschool**-**aged children’s** (**PSAC**) **and school**-**aged children’s** (**SAC**) **co**-**infection status with hookworm**, ***S*** *.* ***stercoralis*** , ***E*** *.* ***vermicularis*** , ***T*** *.* ***trichiura*** , **and**/**or asymptomatic** ***Plasmodium*** **parasitaemia in the Bagamoyo region**, **United Republic of Tanzania**, **and comparison between observed and expected co**-**infection prevalence at the unit of age**-**group**

	Examined children (n)	Observed co-infection (%)	Expected co- infection (%)*	P- value**
**Asymptomatic** ***Plasmodium*** **and** ***E*** ***.*** ***vermicularis*** **infection**				
Infants	167	0.60	0.30	0.413
PSAC	240	1.67	1.46	0.760
SAC	315	5.71	5.44	0.755
All age-groups	722	3.19	2.44	0.156
**Asymptomatic** ***Plasmodium*** **and hookworm infection**				
Infants	204	0.49	0.19	0.338
PSAC	322	0.93	0.86	0.749
SAC	454	2.42	2.28	0.854
All age-groups	980	1.53	1.22	0.329
**Asymptomatic** ***Plasmodium*** **and** ***S*** ***.*** ***stercoralis*** **infection**				
Infants	190	2.11	0.46	0.006
PSAC	308	0.97	0.75	0.715
SAC	438	1.83	1.36	0.345
All age-groups	936	1.60	0.96	0.039
**Asymptomatic** ***Plasmodium*** **and** ***T*** ***.*** ***trichiura*** **infection**				
Infants	204	0.00	0.04	1.000
PSAC	321	0.00	0.25	1.000
SAC	454	0.44	0.61	1.000
All age-groups	979	0.20	0.33	0.760
**Hookworm and** ***E*** ***.*** ***vermicularis*** **infection**				
Infants	155	0.65	0.12	0.181
PSAC	229	2.18	1.93	0.782
SAC	305	3.61	3.23	0.693
All age-groups	689	2.47	1.80	0.138
**Hookworm and** ***S*** ***.*** ***stercoralis*** **infection**				
Infants	188	0.53	0.12	0.216
PSAC	307	0.98	0.59	0.406
SAC	436	1.61	0.90	0.093
All age-groups	931	1.18	0.62	0.038
**Hookworm and** ***T*** ***.*** ***trichiura*** **infection**				
Infants	204	0.00	0.01	1.000
PSAC	321	0.93	0.21	0.022
SAC	454	0.88	0.41	0.100
All age-groups	979	0.72	0.22	0.004
***S*** ***.*** ***stercoralis*** **and** ***E*** ***.*** ***vermicularis*** **infection**				
Infants	144	0.00	0.19	1.000
PSAC	215	1.40	1.48	1.000
SAC	292	1.37	1.67	0.789
All age-groups	651	1.08	1.26	0.841
***S*** ***.*** ***stercoralis*** **and** ***T*** ***.*** ***trichiura*** **infection**				
Infants	188	0.00	0.03	1.000
PSAC	307	0.33	0.17	0.423
SAC	436	0.92	0.24	0.016
All age-groups	931	0.54	0.17	0.018
***E*** ***.*** ***vermicularis*** **and** ***T*** ***.*** ***trichiura*** **infection**				
Infants	155	0.00	0.02	1.000
PSAC	228	0.44	0.53	1.000
SAC	305	1.31	0.93	0.484
All age-groups	688	0.73	0.50	0.361

*Expected co-infection prevalence is the product of the observed infection prevalence of one species (regardless of a co-infection) and the observed infection prevalence of the other species (irrespective of co*-*infection).

**Comparison of observed and expected co-infection proportions, p-value based on a Fisher's Exact-test.

### Association of helminth infections with anthropometric measures, haematology, and parasitic co-infections

Results of the multivariable regression models stratified by helminth infection and age-group are shown in detail in Table 4. After adjusting for potential confounders in multivariable analyses and here only presenting OR higher than 2.00, strongyloidiasis was associated with asymptomatic *Plasmodium* parasitaemia in infants (*S. stercoralis* as outcome: OR: 13.03; 95% CI: 1.34 – 127.23; asymptomatic *Plasmodium* parasitaemia as outcome: OR: 5.75; 95% CI: 1.21 – 27.41). Elevated eosinophil counts in infants were a predictor for *S. stercoralis* (OR: 4.00; 95% CI: 1.10 – 14.58) and hookworm infections (OR: 16.60; CI: 1.39 - 198.32). Infants with a reported anthelmintic treatment in the past 6 months were more likely to be infected with hookworm (OR: 27.91; 95% CI: 5.52 – 141.21).

**Table 4:** **Helminth infections and significantly associated factors according to stepwise backwards multivariable regression analyses in infants**, **preschool**-**aged (PSAC)**, **and school**-**aged children (SAC) from the Bagamoyo district**, **United Republic of Tanzania**

Helminth infection	Age-group	Explanatory variable	n	OR	95% Confidence interval	p- value	Original model run with*
***E*** *.* ***vermicularis***	**all age**-**groups**	Gender	585	0.70	(0.45 - 1.11)	0.127	9,1,2,3,4,6,7,16,17,20
		Age (month)		1.03	(1.02 - 1.05)	<0.001	
		Temperature		1.60	(0.96 - 2.67)	0.071	
		Weight		0.91	(0.82 - 1.00)	0.051	
		Anthelmintic treatment		2.15	(1.22 - 3.79)	0.008	
		Neutrophil counts		1.10	(1.01 - 1.20)	0.034	
	**Infants**	Gender	158	3.72	(0.68 - 20.36)	0.129	9,1,2,3,21
		Age (month)		1.13	(1.01 - 1.27)	0.027	
	**PSAC**	Gender	201	0.51	(0.23 - 1.16)	0.108	9,1,2,3,16
		Anthelmintic treatment		3.60	(1.01 - 12.86)	0.049	
		Neutrophil counts		1.09	(1.00 - 1.19)	0.041	
	**SAC**	Temperature	315	2.21	(1.13 - 4.33)	0.021	9,1,2,3,4
		Anthelmintic treatment		1.78	(0.94 - 3.36)	0.076	
***S*** *.* ***stercoralis***	**all age**-**groups**	Gender	624	0.62	(0.32-1.21)	0.163	11,1,2,3,4,10,12,13,14,19
		Anthelmintic treatment		1.65	(0.80-3.41)	0.178	
		*T. trichiura*		4.13	(1.04-16.52)	0.045	
		Asymptomatic *Plasmodium* parasitaemia		2.10	(0.97-4.51)	0.058	
		Eosinophil counts		2.04	(1.20-3.48)	0.008	
		Platelet counts		1.00	(0.99-1.00)	0.064	
	**Infants**	Gender	132	0.13	(0.01 - 1.38)	0.091	11,1,2,3,6,7,13,14
		Age (month)		1.20	(0.97 - 1.49)	0.086	
		Asymptomatic *Plasmodium* parasitaemia		13.03	(1.34 - 127.23)	0.027	
		Eosinophil counts		4.00	(1.10 - 14.58)	0.036	
	**PSAC**	Light anaemia	303	0.19	(0.04 - 0.82)	0.026	11,1,2,3,21
	**SAC**	*T. trichiura*	290	3.59	(0.80 - 16.08)	0.095	11,1,2,3,4,12,14,19
		Eosinophil counts		2.15	(1.10 - 4.23)	0.026	
		Platelet counts		0.99	(0.98 - 1.00)	0.011	
**Hookworm**	**all age**-**groups**	Temperature	623	1.77	(0.83 - 3.78)	0.143	10,1,2,3,4,6,7,11,12,14,16,20
		Weight		1.12	(1.05 - 1.18)	<0.001	
		*T. trichiura*		3.33	(0.77 - 14.39)	0.107	
		Eosinophil counts		2.30	(1.29 - 4.09)	0.005	
	**Infants**	Anthelmintic treatment	138	27.91	(5.52 - 141.21)	<0.001	10,1,2,3,6,7,14,16,20
		Eosinophil counts		16.60	(1.39 - 198.32)	0.026	
		Basophil counts		0.10	(0.01 - 0.86)	0.036	
		Haemoglobin		1.96	(1.13 - 3.41)	0.016	
	**PSAC**	Gender	272	1.97	(0.72 - 5.38)	0.185	10,1,2,3,4,12,16
		Temperature		3.74	(1.14 - 12.28)	0.030	
		Anthelmintic treatment		0.52	(0.2 - 1.34)	0.174	
		*T. trichiura*		22.78	(4.43 - 117.23)	<0.001	
		Neutrophil counts		1.11	(1.01 - 1.21)	0.03	
	**SAC**	Eosinophil counts	295	2.71	(1.39 - 5.31)	0.004	10,1,2,3,14,27
***T*** *.* ***trichiura***	**all age**-**groups**	Anthelmintic treatment	623	0.12	(0.03 - 0.54)	0.005	12,1,2,3,11,14,18
		*S. stercoralis*		5.18	(1.45 - 18.46)	0.011	
		Eosinophil counts		3.28	(1.69 - 6.36)	0.001	
		Monocyte counts		0.02	(0.00 - 0.33)	0.007	
	**Infants**	Age (month)	55	0.51	(0.31 - 0.85)	0.009	12,1,2,3
	**PSAC**	Hookworm	297	11.53	(2.30 - 57.77)	0.003	12,1,2,3,10,19
		Platelet counts		1.01	(1.00 - 1.01)	0.008	
	**SAC**	Anthelmintic treatment	290	0.08	(0.01 - 0.48)	0.006	12,1,2,3,6,11,14
		*S. stercoralis*		6.63	(1.52 - 28.93)	0.012	
		Eosinophil counts		3.23	(1.73 - 6.02)	0.001	
***Plasmodium*** **parasitaemia**	**all age**-**groups**	Age (month)	952	1.03	(1.02 - 1.04)	<0.001	13,1,2,3,6,7,19,20
		Anthelmintic treatment		0.73	(0.49 - 1.10)	0.137	
		Haemoglobin		0.65	(0.56 - 0.76)	<0.001	
		Platelet counts		1.00	(0.99 - 1.00)	0.012	
	**Infants**	Gender	177	2.88	(0.79 - 10.55)	0.111	13,1,2,3,6,7,11,19
		Weight		1.39	(0.97 - 1.99)	0.071	
		*S. stercoralis*		5.75	(1.21 - 27.41)	0.028	
		Platelet counts		0.99	(0.99 - 1.00)	0.022	
	**PSAC**	Weight	230	1.27	(1.06 - 1.52)	0.010	13,1,2,3,7,18,20
		Haemoglobin		0.49	(0.34 - 0.73)	<0.001	
		Monocyte counts		13.88	(3.42 - 56.30)	<0.001	
	**SAC**	Age (month)	312	1.03	(1.01 - 1.05)	0.004	13,1,2,3,18,20
		Anthelmintic treatment		0.65	(0.36 - 1.19)	0.161	
		Haemoglobin		0.69	(0.53 - 0.90)	0.007	
		Monocyte counts		2.54	(0.77 - 8.37)	0.126	
***Plasmodium*** **parasitaemia**	**<2 years**	Light anaemia	625	1.84	(1.04 - 3.27)	0.037	13,1,2,3,21
		Moderate anaemia		5.36	(2.92 - 9.82)	<0.001	
		Severe anaemia		11.20	(4.20 - 29.86)	<0.001	
		Age (month)		1.04	(1.025 - 1.05)	<0.001	
		Anthelmintic treatment		0.72	(0.47 - 1.10)	0.129	
	**>2 years**	Light anaemia	537	1.57	(0.88 - 2.78)	0.123	13,1,2,3,21
		Moderate anaemia		3.07	(1.96 - 4.81)	<0.001	
		Severe anaemia		7.27	(2.04 - 25.96)	0.002	
		Gender		0.70	(0.47 - 1.04)	0.076	
		Age (month)		1.02	(1.00 - 1.03)	0.021	

*Explanatory variables with significant outcome in the univariable analysis and included in the original model of the multivariate analysis 1: Gender; 2: Age (month); 3: Anthelmintic treatment; 4: Temperature; 5: mid-upper arm circumference (MUAC); 6: Height; 7: Weight; 8: *A. lumbricoides* infection; 9: *E. vermicularis* infection; 10: Hookworm infection; 11: *S. stercoralis* infection; 12: *T. trichiura* infection; 13: Asymptomatic *Plasmodium* parasitaemia; 14: Eosinophil counts; 15: Basophil counts; 16: Neutrophil counts; 17: Lymphocyte counts; 18: Monocyte counts; 19: Platelet counts; 20: Haemoglobin; 21: Anaemia; 22: Malnutrition; 23: Stunting; 24: Thinness; 25: Underweight; 26: Wasting; 27: Fever.

The table shows the significant explanatory variables that remained in the final model with p < 0.2.

Preschool-aged children with increased temperature had higher odds of presenting with a hookworm infection (OR: 3.74; 95% CI: 1.14 – 12.28). Hookworm and *T. trichiura* infections were positively associated in this age-group (hookworm as outcome OR: 22.78%; 95% CI: 4.43 – 117.23; *T. trichiura* as outcome OR: 11.53; 95% CI: 2.30 – 57.77). Elevated monocyte counts were a predictor for asymptomatic *Plasmodium* parasitaemia in PSAC (OR: 13.88; 95% CI 3.42 - 56.30).

School-aged children with *S. stercoralis* infection had higher odds of being co-infected with *T. trichiura* (OR: 6.63; 95% CI: 1.52 – 28.93). In this age-group, children presenting with increased temperature were more likely to be infected with *E. vermicularis* (OR: 2.21; 95% CI: 1.13 – 4.33). Increased eosinophil counts were predictors for infections with *T. trichiura*, hookworm, and *S. stercoralis* (OR: 3.23; 95% CI: 1.73 – 6.02, OR: 2.71; 95% CI: 1.39 – 5.31; and OR: 2.15; 95% CI: 1.10 – 14.58, respectively).

Grouping children according to WHO anaemia thresholds, in children below the age of two years asymptomatic *Plasmodium* parasitaemia was associated with light (OR: 1.84; 95% CI 1.04 – 3.27), moderate (OR: 5.36; 95% CI 2.92 – 9.82), or severe anaemia (OR: 11.20; 4.20 – 29.86). Also children aged older than two years presenting with moderate (OR: 3.07; 95% CI 1.96 – 4.81) or severe anaemia (OR: 7.27; 95% CI 2.04 – 25.96) were more likely to have an asymptomatic *Plasmodium* parasitaemia. In turn, children in the latter age-range presenting with moderate asymptomatic *Plasmodium* parasitaemia were more likely to be anaemic (OR: 2.69; 95% CI: 1.23 – 5.86).

Wasting, underweight, stunting, thinness, fever and microhaematuria were not associated with any helminth infection, asymptomatic *Plasmodium* parasitaemia or anaemia in our study population.

## Discussion

Little is known about the epidemiology and public health importance of *S. stercoralis* and *E. vermicularis* infections in Sub-Saharan Africa. We found that strongyloidiasis and enterobiasis were predominant to other helminthiases in our study population. Noteworthy, already infants, an age-group mostly ignored in studies pertaining to soil-transmitted helminthiases, presented with *S. stercoralis* (5.8%) and *E. vermicularis* infections (4.2%). Prevalences increased with age, and 7.5% and 7.1% of PSAC and SAC, respectively, were infected with *S. stercoralis* and 16.7% and 26.3%, respectively, with *E. vermicularis*.

The prevalences for all helminth infections determined in our study population are relatively low compared to those reported from other settings in the United Republic of Tanzania [36],[55],[56]. The following considerations are offered for explanation: Firstly, over the past decade, Tanzania has successfully implemented a series of helminth control interventions [57],[58], which might have reduced the infections in the population. Indeed, our under five year old children might have received Mebendazole in the frame of the biannual interventions of the Expanded Programme on Immunisation (EPI), which provides vitamin A supplementation, measles vaccination, and deworming in the Bagamoyo District, and the SAC might have been targeted by school-based deworming organized by the Ministry of Health. Secondly, we included infants, PSAC, and SAC aged two months to ten years in our study. The age-prevalence-curve for the major soil-transmitted helminth infections and schistosomiasis increases with age and peaks in children between 8 and 15 years [3],[59]. Our children were hence not yet in the peak age for infection, but an increase in the prevalence of all investigated helminth species with age was indeed observed. Thirdly, the conventional parasitological diagnostic methods that we applied lack sensitivity, particularly when infection intensities are low and only a single faecal sample is examined [9],[60],[61]. While we used duplicate Kato-Katz thick smears and the FLOTAC dual technique on a single stool sample to detect soil-transmitted helminth and *S. mansoni* infections and duplicate urine filtrations from a single urine for *S. haematobium* diagnosis, the true helminth prevalences in our study were likely considerably higher.

*S. stercoralis* infections were associated with eosinophilia in our study population, a condition that is generally attributed to strongyloidiasis [13],[17]. Our study also confirmed results from the neighbouring Zanzibar island with regard to a positive association between *S. stercoralis* and *T. trichiura* infections [55]. Novel is our finding that strongyloidiasis was associated with asymptomatic *Plasmodium* parasitaemia in infants. While the expected prevalence of this co-infection in infants was only 0.5%, the observed prevalence was 2.1%. However, due to the very low number of co-infected infants and multiple testing, these findings must be interpreted with care. If future studies with a larger number of co-infected children confirm our results, it might be reasonable to assume that this tissue invasive helminth species in particular is modulating immunological pathways related to the acquisition, replication, and pathologic sequelae of *Plasmodium* infections. In the worst case, *S. stercoralis* infections in infants may be a contributing factor to the delayed acquisition of clinical immunity to malaria in this highly vulnerable age-group. More indepth-studies on the immunological interplay of strongyloidiasis and malaria are clearly needed.

To date, leading reviews on the global distribution and risk factors for *S. stercoralis* do not mention malaria, and publications summarizing the epidemiology and immunological interplay of helminth and malaria co-infection ignore *S. stercoralis*, respectively. The only study we found assessing *S. stercoralis* infections and malaria was on pregnant women from Uganda, and revealed no association [62]. Moreover, there are only very few publications mentioning *S. stercoralis* infections in infants and PSAC in sub-Saharan Africa, and there are no recent reports assessing strongyloidiasis in any age-group on mainland Tanzania [10],[19],[63]-[65]. Our findings that *S. stercoralis* was infecting a considerable number of young children of our study population from the age of ten months onwards, and was associated with asymptomatic *Plasmodium* parasitaemia, underline the importance of diagnosing this disease. Inclusion of *S. stercoralis* into helminth control programs, as suggested by other research teams, needs to be considered [7],[19],[21].

Enterobiasis was not associated with any specific haematological marker or helminth co-infection, but children of all age-groups with a reported anthelminthic treatment history over the past six months and SAC with an increased temperature had higher odds of an infection. This finding suggests that children with enterobiasis suffer, likely from anal itching and ill-being, and sought treatment for relief. The higher odds of infection might be a sign that either the treatment as administered did not cure the infection or that children got rapidly reinfected, likely due to transmission from additional infected family members [25]. Similarly, infants with reported anthelminthic treatment history had higher odds of hookworm infection in our study. Also hookworm infection can considerably impact on a child’s health and wellbeing [10] and therefore treatment might have been sought. However, infants with hookworm infection who had a reported treatment history might either not have been cured by the treatment or live in a particularly unhygienic environment that favours rapid reinfection. Cure might not have been achieved since, in case mebendazole was administered, cure rates for hookworm are very low [66] or, in case no liquid formulation was available, crushed tablets were difficult to administer to very young children and might not have resulted in complete clearance of infection.

Neither *E. vermicularis* nor hookworm infection was associated with asymptomatic *Plasmodium* parasitaemia in our study. While there are hardly any cross-sectional surveys assessing *E. vermicularis* infections with appropriate diagnostic methods in sub-Saharan Africa, let alone its potential association with malaria, there are multiple studies investigating associations between hookworm and *Plasmodium* infections. In line with our findings, there are studies that did not find an association between hookworm and *Plasmodium* infections [67],[68]. Other studies, however, indicate significant associations between these infectious diseases [38],[62],[69]. The heterogeneous results and potential consequences of co-infections are nicely summarized in recent reviews [27],[28].

Neither *S. stercoralis*, *E. vermicularis*, hookworm, nor any other investigated helminth infection was associated with wasting, underweight, thinness, stunting, or anaemia in our study population. While wasting, underweight, and thinness were rarely seen in the surveyed children and therefore no association could be determined, stunting occurred in 18.5% of SAC and anaemia affected more than half of the examined children. Previous studies conducted in the Kilombero district in Tanzania in the early 1980s had also revealed no association between wasting or stunting and intestinal helminth infection or *Plasmodium* parasitaemia [65]. In that particular time and place the observed substantial differences in the nutritional status of children were related to the lean and post-harvest seasons [70],[71]. The Bagamoyo district, however, is relatively rich in cash-crop production agricultural systems throughout the year, and was one of the four councils in Tanzania, that were not reported to have major food and nutrition insecurity problems in 2011/12 [72]. This might explain why no acute but only chronic signs of malnutrition like stunting were found in our study. Other studies indicated that particularly moderate-to-heavy intensity helminth infections were associated with reduced length-for-age z-score in young infected children from Peru [73], with decreased weight-for-age z-scores in SAC from Honduras [74], and with stunting in SAC from China [75]. The occurrence of mostly light helminth infection intensities in our study might hence explain why no association was found.

Anaemia was related with asymptomatic *Plasmodium* parasitaemia but not with helminthiases in infants, PSAC, and SAC in our study. It is widely acknowledged that *Plasmodium* infections, also if low-grade and asymptomatic as a result of semi-immunity, contribute to the development of anaemia [76]. Also hookworm infections have been shown to contribute to anaemia, particularly in children and women of childbearing age, and when infection intensities were moderate-to-heavy [3],[77]-[80]. Also here, the reason for not finding any association of helminth infections with anaemia might be due to the mostly light infection intensities found in our study population. Clearly, the low prevalence of helminth infections and specifically of moderate and high infection intensities, resulting in a lack of statistical power to determine effects on nutritional aspects including anaemia and stunting, are a limitation of this study. Moreover, we did not assess additional reasons for malnutrition and anaemia, such as restricted access to micro-nutrients, agricultural and dietary practices, food security, or social, political, and economic determinants, which might have biased our results.

Elevated eosinophil counts were strongly associated with hookworm (OR: 16.6) and *S. stercoralis* (OR: 4.0) infections in infants and with *T. trichiura* (OR: 3.2), hookworm (OR: 2.7), and *S. stercoralis* (OR: 2.2) infections in SAC. Marked eosinophilia is considered a common marker for early hookworm and *S. stercoralis* infections, and is explained by the immune reaction to the worm larvae, which migrate through the body tissues to reach their destination in the lungs and in the gastrointestinal tract [13]. The eosinophilia caused by *T. trichiura* is reported to be mostly mild [13]. The hematology values of our study population were only partially in line with reference values derived from a similar study population based in the Kilimanjaro district [51]. Considerably higher neutrophil counts were found in almost a quarter and elevated monocyte, eosinophil, and basophil counts in more than a third of all participants of all age-groups in our study children. While higher eosinophil counts were associated with specific helminth species infections and monocyte counts were associated with asymptomatic *Plasmodium* parasitemia in some age-groups, the latter being a common sign of acute malaria [81],[82], there might be additional underlying reasons such as viral or bacterial infections causing monocytosis or allergies and other diseases causing eosinophilia [13],[83]. Elevated basophil counts were not associated with helminth or asymptomatic *Plasmodium* infections in our study, confirming that it is not a useful clinical marker for the evaluation of suspected parasitic disease [84]. Moreover, considering the unpublished reference values used in the IHI-BRTC laboratory, the normal range of basophils at the IHI-BRTC is 0.0-0.3 per 10^9^/L and thus much higher than the Kilimanjaro values [51] and rather resembling the pediatric reference values reported for Uganda [85]. A limitation of our study is that no other infections or diseases that might have caused elevated or decreased blood cell counts were investigated and there is a clear need for the establishment of standardized hematological reference values for the Bagamoyo area.

## Conclusions

The results of our cross-sectional study showed that *E. vermicularis* and *S. stercoralis* were moderately prevalent in young children from rural coastal Tanzania. A considerable number of infants were infected and prevalences increased with children’s age. Our data can contribute to inform yet missing global burden of disease and prevalence estimates for strongyloidiasis and enterobiasis. The association between *S. stercoralis* and asymptomatic *Plasmodium* parasitaemia found in infants of our study population warrants further investigations.

## Authors’ original submitted files for images

Below are the links to the authors’ original submitted files for images. Authors’ original file for figure 1 Authors’ original file for figure 2

## Abbreviations

CI: Confidence interval
CRF: Case report form
DALYs: Disability adjusted life years
EKBB: Ethikkomission beider Basel
EPG: Eggs per gram of stool
FEC: Faecal egg count
HIV-AIDS: Human immunodeficiency virus-acquired immune deficiency syndrome
ICT: Immunochromatic test
IHI-BRTC: Ifakara Health Institute-Bagamoyo Research and Training Centre
MUAC: Mid-upper arm circumference
NIMR: National Institution for Medical Research of Tanzania
OR: Odds ratio
PanACEA: Pan African Collaboration for the Evaluation of Antituberculosis Antibiotics
PSAC: Preschool-aged children
SAC: School-aged children
Swiss TPH: Swiss Tropical and Public Health Institute
TB: Tuberculosis
UNICEF: United Nations Children's Fund
VHCW: Village health care worker
WHO: World Health Organization

## References

[1] Utzinger J, Becker SL, Knopp S, Blum J, Neumayr AL, Keiser J, Hatz CF (2012). Neglected tropical diseases: diagnosis, clinical management, treatment and control. Swiss Med Wkly.

[2] Knopp S, Steinmann P, Keiser J, Utzinger J (2012). Nematode infections: soil-transmitted helminths and *Trichinella*. Infect Dis Clin North Am.

[3] Bethony J, Brooker S, Albonico M, Geiger SM, Loukas A, Diemert D, Hotez PJ (2006). Soil-transmitted helminth infections: ascariasis, trichuriasis, and hookworm. Lancet.

[4] Pullan RL, Smith JL, Jasrasaria R, Brooker SJ (2014). Global numbers of infection and disease burden of soil transmitted helminth infections in 2010. Parasit Vectors.

[5] Hotez PJ, Alvarado M, Basáñez MG, Bolliger I, Bourne R, Boussinesq M, Brooker SJ, Brown AS, Buckle G, Budke CM, Carabin H, Coffeng LE, Fevre EM, Fürst T, Halasa YA, Jasrasaria R, Johns NE, Keiser J, King CH, Lozano R, Murdoch ME, O'Hanlon S, Pion SD, Pullan RL, Ramaiah KD, Roberts T, Shepard DS, Smith JL, Stolk WA, Undurraga EA (2014). The Global Burden of Disease Study 2010: interpretation and implications for the neglected tropical diseases. PLoS Negl Trop Dis.

[6] Murray CJL, Vos T, Lozano R, Naghavi M, Flaxman AD, Michaud C, Ezzati M, Shibuya K, Salomon JA, Abdalla S, Aboyans V, Abraham J, Ackerman I, Aggarwal R, Ahn SY, Ali MK, Alvarado M, Anderson HR, Anderson LM, Andrews KG, Atkinson C, Baddour LM, Bahalim AN, Barker-Collo S, Barrero LH, Bartels DH, Basáñez MG, Baxter A, Bell ML, Benjamin EJ (2012). Disability-adjusted life years (DALYs) for 291 diseases and injuries in 21 regions, 1990-2010: a systematic analysis for the Global Burden of Disease Study 2010. Lancet.

[7] Krolewiecki AJ, Lammie P, Jacobson J, Gabrielli AF, Levecke B, Socias E, Arias LM, Sosa N, Abraham D, Cimino R, Echazu A, Crudo F, Vercruysse J, Albonico M (2013). A public health response against *Strongyloides stercoralis*: time to look at soil-transmitted helminthiasis in full. PLoS Negl Trop Dis.

[8] Olsen A, van Lieshout L, Marti H, Polderman T, Polman K, Steinmann P, Stothard R, Thybo S, Verweij JJ, Magnussen P (2009). Strongyloidiasis – the most neglected of the neglected tropical diseases?. Trans R Soc Trop Med Hyg.

[9] Knopp S, Salim N, Schindler T, Karagiannis Voules DA, Abduhl U, Rothen J, Lweno O, Mohammed AS, Genton B, Daubenberger C (2014). Diagnostic accuracy of Kato-Katz, FLOTAC, Baermann and PCR methods for the detection of light intensity hookworm and *Strongyloides stercoralis*infections in Tanzania. Am J Trop Med.

[10] Becker SL, Sieto B, Silue KD, Adjossan L, Kone S, Hatz C, Kern WV, N'Goran EK, Utzinger J (2011). Diagnosis, clinical features, and self-reported morbidity of *Strongyloides stercoralis*and hookworm infection in a co-endemic setting. PLoS Negl Trop Dis.

[11] Montes M, Sawhney C, Barros N (2010). *Strongyloides stercoralis*: there but not seen. Curr Opin Infect Dis.

[12] Greaves D, Coggle S, Pollard C, Aliyu SH, Moore EM (2013). *Strongyloides stercoralis*infection. BMJ.

[13] Leder K, Weller PF (2000). Eosinophilia and helminthic infections. Baillieres Best Pract Res Clin Haematol.

[14] Grove DI (1996). Human strongyloidiasis. Adv Parasitol.

[15] Khieu V, Srey S, Schar F, Muth S, Marti H, Odermatt P (2013). *Strongyloides stercoralis*is a cause of abdominal pain, diarrhea and urticaria in rural Cambodia. BMC Res Notes.

[16] Ardic N (2009). [An overview of *Strongyloides stercoralis*and its infections]. Mikrobiyol Bul.

[17] Nuesch R, Zimmerli L, Stockli R, Gyr N, Hatz CFR (2005). Imported strongyloidosis: a longitudinal analysis of 31 cases. J Travel Med.

[18] Segarra-Newnham M (2007). Manifestations, diagnosis, and treatment of *Strongyloides stercoralis*infection. Ann Pharmacother.

[19] Schär F, Trostdorf U, Giardina F, Khieu V, Muth S, Marti H, Vounatsou P, Odermatt P (2013). *Strongyloides stercoralis*: global distribution and risk factors. PLoS Negl Trop Dis.

[20] Utzinger J, Raso G, Brooker S, De Savigny D, Tanner M, Ornbjerg N, Singer BH, N'Goran EK (2009). Schistosomiasis and neglected tropical diseases: towards integrated and sustainable control and a word of caution. Parasitology.

[21] Bisoffi Z, Buonfrate D, Montresor A, Requena-Mendez A, Munoz J, Krolewiecki AJ, Gotuzzo E, Mena MA, Chiodini PL, Anselmi M, Moreira J, Albonico M (2013). *Strongyloides stercoralis*: a plea for action. PLoS Negl Trop Dis.

[22] St Georgiev V (2001). Chemotherapy of enterobiasis (oxyuriasis). Expert Opin Pharmacother.

[23] Arca MJ, Gates RL, Groner JI, Hammond S, Caniano DA (2004). Clinical manifestations of appendiceal pinworms in children: an institutional experience and a review of the literature. Pediatr Surg Int.

[24] Burkhart CN, Burkhart CG (2005). Assessment of frequency, transmission, and genitourinary complications of enterobiasis (pinworms). Int J Dermatol.

[25] Cook GC (1994). *Enterobius vermicularis*infection. Gut.

[26] Fry GF, Moore JG (1969). *Enterobius vermicularis*: 10,000-year-old human infection. Science.

[27] Nacher M (2011). Interactions between worms and malaria: good worms or bad worms?. Malar J.

[28] Adegnika AA, Kremsner PG (2012). Epidemiology of malaria and helminth interaction: a review from 2001 to 2011. Curr Opin HIV AIDS.

[29] Rafi W, Ribeiro-Rodrigues R, Ellner JJ, Salgame P (2012). Coinfection-helminthes and tuberculosis. Curr Opin HIV AIDS.

[30] Walson JL, Herrin BR, John-Stewart G (2009). Deworming helminth co-infected individuals for delaying HIV disease progression. Cochrane Database Syst Rev.

[31] Secor WE (2012). The effects of schistosomiasis on HIV/AIDS infection, progression and transmission. Curr Opin HIV AIDS.

[32] Webb EL, Ekii AO, Pala P (2012). Epidemiology and immunology of helminth-HIV interactions. Curr Opin HIV AIDS.

[33] Knopp S, Becker S, Ingram K, Keiser J, Utzinger J (2013). Diagnosis and treatment of schistosomiasis in children in the era of intensified control. Expert Rev Anti Infect Ther.

[34] Moreau E, Chauvin A (2010). Immunity against helminths: interactions with the host and the intercurrent infections. J Biomed Biotechnol.

[35] McSorley HJ, Hewitson JP, Maizels RM (2013). Immunomodulation by helminth parasites: defining mechanisms and mediators. Int J Parasitol.

[36] Kinung'hi SM, Magnussen P, Kaatano GM, Kishamawe C, Vennervald BJ (2014). Malaria and helminth co-infections in school and preschool children: a cross-sectional study in Magu district, north-western Tanzania. PLoS One.

[37] Salgame P, Yap GS, Gause WC (2013). Effect of helminth-induced immunity on infections with microbial pathogens. Nat Immunol.

[38] Righetti AA, Glinz D, Adiossan LG, Koua AY, Niamke S, Hurrell RF, Wegmuller R, N'Goran EK, Utzinger J (2012). Interactions and potential implications of *Plasmodium falciparum*-hookworm coinfection in different age groups in south-central Côte d'Ivoire. PLoS Negl Trop Dis.

[39] Mwangi TW, Bethony JM, Brooker S (2006). Malaria and helminth interactions in humans: an epidemiological viewpoint. Ann Trop Med Parasitol.

[40] NBS & OCGS (2013). 2012 Population and Housing Survey of the United Republic of Tanzania. Population Distribution by Administrative Areas.

[41] NBS & CRCO (2007). United Republic of Tanzania Coast Region Socio-Economic Profile.

[42] Greenwood BM, Armstrong JR (1991). Comparison of two simple methods for determining malaria parasite density. Trans R Soc Trop Med Hyg.

[43] NMCP, WHO, IHI, KEMRI (2013). An Epidemiological Profile of Malaria and its Control in Mainland Tanzania. Report Funded by Roll Back Malaria and Department for International Development-UK.

[44] García LS, Bruckner DA (2001). Diagnostic Medical Parasitology.

[45] Katz N, Chaves A, Pellegrino J (1972). A simple device for quantitative stool thick-smear technique in schistosomiasis mansoni. Rev Inst Med Trop Sao Paulo.

[46] Peters PA, Mahmoud AA, Warren KS, Ouma JH, Siongok TK (1976). Field studies of a rapid, accurate means of quantifying *Schistosoma haematobium*eggs in urine samples. Bull World Health Organ.

[47] Cringoli G, Rinaldi L, Maurelli MP, Utzinger J (2010). FLOTAC: new multivalent techniques for qualitative and quantitative copromicroscopic diagnosis of parasites in animals and humans. Nat Protoc.

[48] Mogeni P, Twahir H, Bandika V, Mwalekwa L, Thitiri J, Ngari M, Toromo C, Maitland K, Berkley JA (2011). Diagnostic performance of visible severe wasting for identifying severe acute malnutrition in children admitted to hospital in Kenya. Bull World Health Organ.

[49] WHO & UNICEF (2009). WHO Child Growth Standards and the Identification of Severe Acute Malnutrition in Infants and Children.

[50] WHO (2011). Haemoglobin Concentrations for the Diagnosis of Anaemia and Assessment of Severity.

[51] Buchanan AM, Muro FJ, Gratz J, Crump JA, Musyoka AM, Sichangi MW, Morrissey AB, M'Rimberia JK, Njau BN, Msuya LJ, Bartlett JA, Cunningham CK (2010). Establishment of haematological and immunological reference values for healthy Tanzanian children in Kilimanjaro Region. Trop Med Int Health.

[52] Kohl KS, Marcy SM, Blum M, Connell Jones M, Dagan R, Hansen J, Nalin D, Rothstein E (2004). Brighton Collaboration Fever Working G: fever after immunization: current concepts and improved future scientific understanding. Clin Infect Dis.

[53] Montresor A, Crompton DWT, Hall A, Bundy DAP, Savioli L (1998). Guidelines for the Evaluation of Soil-Transmitted Helminthiasis and Schistosomiasis at Community Level.

[54] Raso G, Vounatsou P, Singer BH, N'Goran EK, Tanner M, Utzinger J (2006). An integrated approach for risk profiling and spatial prediction of *Schistosoma mansoni*-hookworm coinfection. Proc Natl Acad Sci U S A.

[55] Knopp S, Mohammed KA, Stothard JR, Khamis IS, Rollinson D, Marti H, Utzinger J (2010). Patterns and risk factors of helminthiasis and anemia in a rural and a peri-urban community in Zanzibar, in the context of helminth control programs. PLoS Negl Trop Dis.

[56] Tatala SR, Kihamia CM, Kyungu LH, Svanberg U (2008). Risk factors for anaemia in schoolchildren in Tanga Region. Tanzania Tanzan J Health Res.

[57] Nyhus Dhillon C, Subramaniam H, Mulokozi G, Rambeloson Z, Klemm R (2013). Overestimation of vitamin a supplementation coverage from district tally sheets demonstrates importance of population-based surveys for program improvement: lessons from Tanzania. PLoS One.

[58] Mwakitalu ME, Malecela MN, Mosha FW, Simonsen PE (2014). Urban schistosomiasis and soil transmitted helminthiases in young school children in Dar es Salaam and Tanga, Tanzania, after a decade of anthelminthic intervention. Acta Trop.

[59] Gryseels B (2012). Schistosomiasis. Infect Dis Clin North Am.

[60] Knopp S, Mgeni AF, Khamis IS, Steinmann P, Stothard JR, Rollinson D, Marti H, Utzinger J (2008). Diagnosis of soil-transmitted helminths in the era of preventive chemotherapy: effect of multiple stool sampling and use of different diagnostic techniques. PLoS Negl Trop Dis.

[61] Jeandron A, Abdyldaieva G, Usubalieva J, Ensink JH, Cox J, Matthys B, Rinaldi L, Cringoli G, Utzinger J (2010). Accuracy of the Kato-Katz, adhesive tape and FLOTAC techniques for helminth diagnosis among children in Kyrgyzstan. Acta Trop.

[62] Hillier SD, Booth M, Muhangi L, Nkurunziza P, Khihembo M, Kakande M, Sewankambo M, Kizindo R, Kizza M, Muwanga M, Elliott AM (2008). *Plasmodium falciparum*and helminth coinfection in a semi urban population of pregnant women in Uganda. J Infect Dis.

[63] Stothard JR, Pleasant J, Oguttu D, Adriko M, Galimaka R, Ruggiana A, Kazibwe F, Kabatereine NB (2008). *Strongyloides stercoralis*: a field-based survey of mothers and their preschool children using ELISA, Baermann and Koga plate methods reveals low endemicity in western Uganda. J Helminthol.

[64] Joyce T, McGuigan KG, Elmore-Meegan M, Conroy RM (1996). Prevalence of enteropathogens in stools of rural Maasai children under five years of age in the Maasailand region of the Kenyan Rift Valley. East Afr Med J.

[65] Tanner M, Burnier E, Mayombana C, Betschart B, de Savigny D, Marti HP, Suter R, Aellen M, Lüdin E, Degrémont AA (1987). Longitudinal study on the health status of children in a rural Tanzanian community: parasitoses and nutrition following control measures against intestinal parasites. Acta Trop.

[66] Keiser J, Utzinger J (2008). Efficacy of current drugs against soil-transmitted helminth infections: systematic review and meta-analysis. JAMA.

[67] Mazigo HD, Kidenya BR, Ambrose EE, Zinga M, Waihenya R (2010). Association of intestinal helminths and *P. falciparum*infections in co-infected school children in northwest Tanzania. Tanzan J Health Res.

[68] Shapiro AE, Tukahebwa EM, Kasten J, Clarke SE, Magnussen P, Olsen A, Kabatereine NB, Ndyomugyenyi R, Brooker S (2005). Epidemiology of helminth infections and their relationship to clinical malaria in southwest Uganda. Trans R Soc Trop Med Hyg.

[69] Boel M, Carrara VI, Rijken M, Proux S, Nacher M, Pimanpanarak M, Paw MK, Moo O, Gay H, Bailey W, Singhasivanon P, White NJ, Nosten F, McGready R (2010). Complex interactions between soil-transmitted helminths and malaria in pregnant women on the Thai-Burmese border. PLoS Negl Trop Dis.

[70] Tanner M, Lukmanji Z (1987). Food consumption patterns in a rural Tanzanian community (Kikwawila village, Kilombero District, Morogoro Region) during lean and post-harvest season. Acta Trop.

[71] Tanner M, de Savigny D (1987). Monitoring of community health status: experience from a case study in Tanzania. Acta Trop.

[72] MUCHALI (2012). Comprehensive Food Security and Nutrition Assessment Report of the April, 2012 Main (Masika) Season.

[73] Gyorkos TW, Maheu-Giroux M, Casapia M, Joseph SA, Creed-Kanashiro H (2011). Stunting and helminth infection in early preschool-age children in a resource-poor community in the Amazon lowlands of Peru. Trans R Soc Trop Med Hyg.

[74] Sanchez AL, Gabrie JA, Usuanlele MT, Rueda MM, Canales M, Gyorkos TW (2013). Soil-transmitted helminth infections and nutritional status in school-age children from rural communities in Honduras. PLoS Negl Trop Dis.

[75] Shang Y, Tang LH, Zhou SS, Chen YD, Yang YC, Lin SX (2010). Stunting and soil-transmitted-helminth infections among school-age pupils in rural areas of southern China. Parasit Vectors.

[76] Kurtzhals JA, Addae MM, Akanmori BD, Dunyo S, Koram KA, Appawu MA, Nkrumah FK, Hviid L (1999). Anaemia caused by asymptomatic *Plasmodium falciparum*infection in semi-immune African schoolchildren. Trans R Soc Trop Med Hyg.

[77] Smith JL, Brooker S (2010). Impact of hookworm infection and deworming on anaemia in non-pregnant populations: a systematic review. Trop Med Int Health.

[78] Brooker S, Hotez PJ, Bundy DAP (2008). Hookworm-related anaemia among pregnant women: a systematic review. PLoS Negl Trop Dis.

[79] Dreyfuss ML, Stoltzfus RJ, Shrestha JB, Pradhan EK, LeClerq SC, Khatry SK, Shrestha SR, Katz J, Albonico M, West KP (2000). Hookworms, malaria and vitamin A deficiency contribute to anemia and iron deficiency among pregnant women in the plains of Nepal. J Nutr.

[80] Stoltzfus RJ, Chwaya HM, Montresor A, Albonico M, Savioli L, Tielsch JM (2000). Malaria, hookworms and recent fever are related to anemia and iron status indicators in 0- to 5-y old Zanzibari children and these relationships change with age. J Nutr.

[81] Antonelli LR, Leoratti FM, Costa PA, Rocha BC, Diniz SQ, Tada MS, Pereira DB, Teixeira-Carvalho A, Golenbock DT, Goncalves R, Gazzinelli RT (2014). The CD14 + CD16+ inflammatory monocyte subset displays increased mitochondrial activity and effector function during acute *Plasmodium vivax*malaria. PLoS Pathog.

[82] Halim NKD, Ajayi OI, Oluwafemi F (2002). Monocytosis in acute malaria infection. Niger J Clin Pract.

[83] Schulte C, Krebs B, Jelinek T, Nothdurft HD, von Sonnenburg F, Loscher T (2002). Diagnostic significance of blood eosinophilia in returning travelers. Clin Infect Dis.

[84] Mitre E, Nutman TB (2003). Lack of basophilia in human parasitic infections. Am J Trop Med Hyg.

[85] Lugada ES, Mermin J, Kaharuza F, Ulvestad E, Were W, Langeland N, Asjo B, Malamba S, Downing R (2004). Population-based hematologic and immunologic reference values for a healthy Ugandan population. Clin Diagn Lab Immunol.

