# Supplementary figures and images for: Enterobiasis and strongyloidiasis and associated co-infections and morbidity markers in infants, preschool- and school-aged children from rural coastal Tanzania: a cross-sectional study
Source: BMC Infect Dis. 2014 Dec 9;14:644. doi: 10.1186/s12879-014-0644-7 (PMC4271451; doi:10.1186/s12879-014-0644-7)

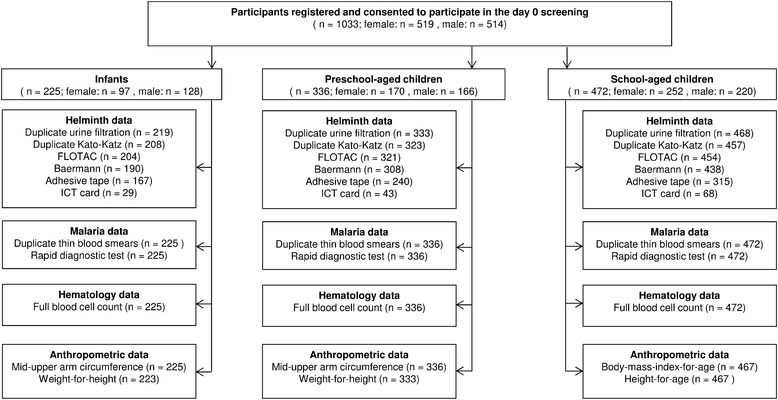

Supplement: Supplementary file 1 — Authors’ original file for figure 1 [file 12879_2014_644_MOESM1_ESM.gif]

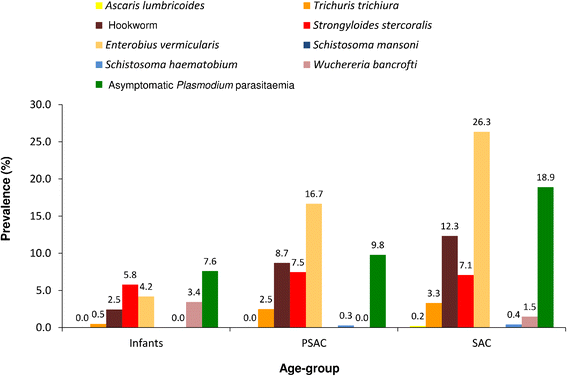

Supplement: Supplementary file 2 — Authors’ original file for figure 2 [file 12879_2014_644_MOESM2_ESM.gif]
